# Spatio-Temporal Variation of Lung Cancer in Crete, 1992–2013. Economic or Health Crisis?

**DOI:** 10.3390/ijerph191912161

**Published:** 2022-09-26

**Authors:** Dimitra Sifaki-Pistolla, Vasiliki Eirini Chatzea, Enkeleint A. Mechili, Filippos Koinis, Vassilis Georgoulias, Christos Lionis, Nikos Tzanakis

**Affiliations:** Cancer Registry of Crete, School of Medicine, University of Crete, 700 13 Heraklion, Greece

**Keywords:** lung cancer, spatial epidemiology, cancer surveillance, economic crisis, deprivation, global health

## Abstract

(1) Background: This is the first population-based study in Greece, with the aim to measure the changing trends of lung cancer (LC) and the associated risk factors before and after the economic crisis. Among the main objectives were the identification of LC hot spots and high-risk areas; (2) Methods: The study was conducted in Crete, the biggest island in Greece. Data (5057 LC cases) were collected from the Cancer Registry of Crete (CRC). The age-standardized incidence and mortality rates (ASIR, ASMR/100,000/year) were estimated, while additional indexes were used, including the adjusted Charlson’s comorbidity index (CCI%), the deprivation index (HPI-2), and the exposure to outdoor air pollution (OAP). The analysis was performed for two time periods (Period A: 1992–2008; Period B: 2009–2013); (3) Results: ASIR presented a significant increase during the economic crisis, while an even higher increase was observed in ASMR (Period A: ASMR = 30.5/100,000/year; Period B: ASMR = 43.8/100,000/year; *p* < 0.001). After 2009, a significant increase in the observed LC hot spots was identified in several sub-regions in Crete (*p* = 0.04). The risk of LC mortality increased even more for smokers (RR = 5.7; 95%CI = 5.2–6.3) and those living in highly deprived geographical regions (RR = 5.4; 95%CI = 5.1–5.8) during the austerity period. The multiple effect of LC predictors resulted in adjusted RRs ranging from 0.7 to 5.7 within the island (*p* < 0.05); (4) Conclusions: The increased LC burden after the onset of the economic crisis, along with a changing pattern of LC predictors stressed the urgent need of geographically oriented interventions and cancer control programs focusing on the most deprived or vulnerable population groups.

## 1. Introduction

The global financial crisis has posed major threats and challenges to multiple societal, political, and health aspects of daily life [1,2]. Since 2008, Europe and mainly Greece have been experiencing one of the most severe financial crises in the recent history, with direct effects on the Greek healthcare system and the community’s health [2,3]. There are numerous studies in the recent literature that have reported the adverse effects of the economic crisis and the constantly increasing unmet health needs of people living in austerity [4,5,6,7,8,9]. Effects of this crisis are mixed and seem to have engulfed the more vulnerable population groups (uninsured, homeless, chronic patients, multi-morbid and frail individuals, and others) [10]. 

Furthermore, several concerns regarding ordinary people’s health have been raised since it’s still unclear to what extend and toward which direction lifestyle and health behavior’s change. Recessions are considered complex events that can affect a person’s health and behavior via several potentially opposing mechanisms. While recessions have negative effects on mental health, it has been proven that they may reduce mortality rates. Nevertheless, there is a lack of consistency between different individual and country level health impact findings [11]. Cancer is among the chronic diseases that tend to be affected during recession periods [12,13,14,15]. Lung cancer (LC) in particular is the most common example of disease-specific rates that present increasing trends in population groups living in austerity [16]. This is mainly due to the limited access in medication or treatment, the difficulties in accessing healthcare services for early diagnosis and the increase in lifestyle risk factors, such as smoking [16,17]. LC survival has been found to be directly affected by poverty and socioeconomic (SES) factors, with approximately 22% of the poorest patients surviving cancer and 45% of the richest population groups surviving for at least 5 years after diagnosis [17].

Northern European countries, such as Greece, Italy, and Spain, are among the countries that have been significantly affected by the economic crisis and have presented evidently increased high poverty rates [18]. Similar observations have been made in Crete, the biggest island in Greece that has undergone several changes after the onset of the economic crisis. A significant percentage of Cretans have long and heavy smoking history, especially in some rural regions where SES is also very low [19,20]. Similar trends are often observed within the major urban centers in Crete where the extensive use of smoking and the relatively high exposure to outdoor air pollution (OAP) are visible, especially during the austerity period. In addition to that, many studies have reported the poor living conditions of multi-morbid patients in rural Crete [20], the neglected LC screening and follow-up, the income cuts and the barriers for medication and therapy [5], and the overall increase in LC incidence and mortality rates in Crete [21].

Furthermore, two previous studies of the Cancer Registry of Crete (CRC) have outlined the joint effect of multiple risk factors to LC rates in Crete, including tobacco use, OAP exposure and multiple morbidities [21,22]. In addition, Lazaridis et al. have found that exposure to OAP and smoking prevalence are higher in regions with higher poverty or deprivation rates [23]. Therefore, the CRC has raised several research questions about the impact of the economic crisis on LC incidence, mortality, and survival, as well as on the potential change in several behavioral, demographic, and environmental risk factors that are related to LC. To that end, the following objectives were set: measure the changing trends of LC and the associated risk factors before and after the economic crisis, identify potential hot spots among the different geographical regions in Crete and the potential LC predictors before and after the economic crisis, and assess the overall risk of LC mortality within the island of Crete.

## 2. Materials and Methods

### 2.1. Setting and Study Population

The current study was conducted in Crete, the biggest island in Greece (approximately 590,000 permanent residents). Crete has four counties, while it is comprised of nineteen municipalities: manly urban, rural, and semi-urban [21,22]. Furthermore, 5057 LC cases were obtained and followed-up from 1992 to 2013 from the CRC’s database (www.crc.uoc.gr). Additionally, there were 3599 LC deaths. LC data were coded according to the international classification system for oncology (ICD10-O-2), while information on patients’ demographic profile, personal and family medical history, and lifestyle factors (smoking habits, alcohol consumption) were also included in the final dataset. 

Three major inclusion criteria were determined as follows: (1) cases with primary LC, (2) individuals that have been residing in Crete for at least the past 10 years, (3) a histologically or cytologically confirmed diagnosis of lung cancer. In addition, several quality checks were performed following the Bray and Parkin guidelines for cancer registry’s quality checks [24]. The CRC achieved very high-quality rates: completeness = 98%, validity/accuracy = 97%, and timeliness = 99%. Data are considered “complete” when they fulfill expectations of comprehensiveness. The term “accuracy” refers to the degree to which information accurately reflects reality, while to be “valid” information should follow a specific format. The data quality dimension of “timeliness” measures whether the information is available to the user right when it’s needed. Further information of the CRC data collection and quality control processes is presented in Sifaki-Pistolla et al., 2016 [21]. 

### 2.2. Incidence and Mortality Rates and Other Indexes

The age-standardize incidence and mortality rates (ASIR and ASMR) were estimated based on the European standard population of 2011 and expressed as number of LC new cases/deaths per 100,000 per year. Additional indexes were used to assess several associated risk factors including socioeconomic, clinical, environmental, and lifestyle parameters, as described below. 

The human poverty index (HPI-2) for high income countries was used to assess poverty and socioeconomic status per municipality in Crete at two time periods (Period A: 1992–2008; Period B: 2009–2013). The HPI concentrates on deprivation in three core components of human life already reflected in the overall development index: longevity, knowledge, and standard of living. The HPI included the following criteria: (a) probability at birth of not surviving to age 60 (%), (b) people lacking functional literacy skills (%), (c) long-term unemployment (%), and (d) population below 50% of median income (%) [25]. The HPI is derived separately for developing countries (HPI-1) and a group of select high-income OECD countries (HPI-2) to better reflect socio-economic differences [25,26]. In the current study, the HPI-2 measured using the median value and the variation rate before and after the economic crisis.

Furthermore, we used the Charlson’s comorbidity index (CCI) to estimate risk of death from comorbid disease for use in longitudinal studies [27]. The CCI is a simple, readily applicable, and valid method that has been extensively used in cancer research [28]. Each condition is assigned a score of 1, 2, 3, or 6, depending on the risk of dying associated with each one, while scores are summed to provide a total score of CCI. Clinical conditions and associated scores are as follows: 1 each: Myocardial infarct, congestive heart failure, peripheral vascular disease, dementia, cerebrovascular disease, chronic lung disease, connective tissue disease, ulcer, chronic liver disease, diabetes; 2 each: Hemiplegia, moderate or severe kidney disease, diabetes with end organ damage, tumor, leukemia, lymphoma; 3 each: Moderate or severe liver disease; 6 each: Malignant tumor, metastasis, AIDS [27,28].

In addition, exposure to outdoor air pollution (OAP) was assessed by measuring PM2.5, between 2.5 μm and 10 μm (PM2.5–10), PM10, PM2.5 absorbance (black carbon measure), nitrogen dioxide (NO2), and nitrogen oxides (NOx). The OAP indicators were collected from two different sources (the European Environment Agency database and primary field measurements), while further details on the results, the methodological and statistical framework have already been reported by a previous study of the CRC [22]. 

### 2.3. Statistical Analysis

The analysis was performed in STATA (v.16, StataCorp LLC, College Station, TX, USA) and ArcMap (v.10.3.1 ESRI, Redlands, CA, USA) software programs, while all tests were conducted at a = 0.05. LC cases were aggregated at municipality level based on the place of residence during the time of diagnosis (for at least the last 10 years). Hotspots analysis (Getis-Ord Gi*) was performed to identify statistically significant spatial clusters of high values (hot spots) and low values (cold spots) before and after the economic crisis [29]. In addition, two Bayesian multivariate regression models were used in order to estimate the risk of dying from LC before and after the economic crisis; testing for multiple covariates and adjusting for age, gender, stage at diagnosis, and place of residence. Furthermore, a final regression model was developed using the statistically significant predictors of LC mortality to assess the current risk areas in Crete [30].

### 2.4. Ethical Approval

The CRC holds a license from the Hellenic Data Protection Authority (Protocol number: 960/11-08-2009) and has adopted the rules for collecting, managing, and processing sensitive and personal data. All information was recorded using a cryptographic coding system in accordance with the federal law principles and stored in the CMS server. No personal or individual-level data are or will be published. 

## 3. Results

### 3.1. Changing Trends of LC and Associated Factors before and after the Economic Crisis

LC incidence and mortality rates as well as most of the cancer-related rates varied significantly after the onset of the economic crisis (Table 1). ASIR presented a significant increase of 9.8 from Period A to Period B (*p* = 0.01), ranging from 37.9/100,000/year to 47.8/100,000/year. An even higher increase was observed in ASMR that presented a variation rate of 13.3 (Period A: ASMR = 30.5/100,000/year; Period B: ASMR = 43.8/100,000/year; *p* < 0.001). A lower but statistically significant decrease was observed in five-year net-survival (%) (variation rate = −2.5; *p* = 0.04). Males presented decreasing trends during the austerity period (Variation rate = −9.3) contrary to females that increased rapidly (variation rate = 9.4). Furthermore, the median age at year of diagnosis increased significantly from 68 to 77 years (*p* < 0.001). The adjusted CCI% increased slightly (variation rate = 1; *p* = 0.04), while a significant increase was also observed in smokers (variation rate = 7.4; *p* = 0.02) and alcohol consumers (variation rate = 9.4; *p* = 0.02). In addition, exposure to OAP (variation rate = 9.8; *p* < 0.001) and the median deprivation index (variation rate = 11.7; *p* < 0.001) presented major increasing trends.

### 3.2. LC Hot Spots before and after the Economic Crisis

**Figure 1:**
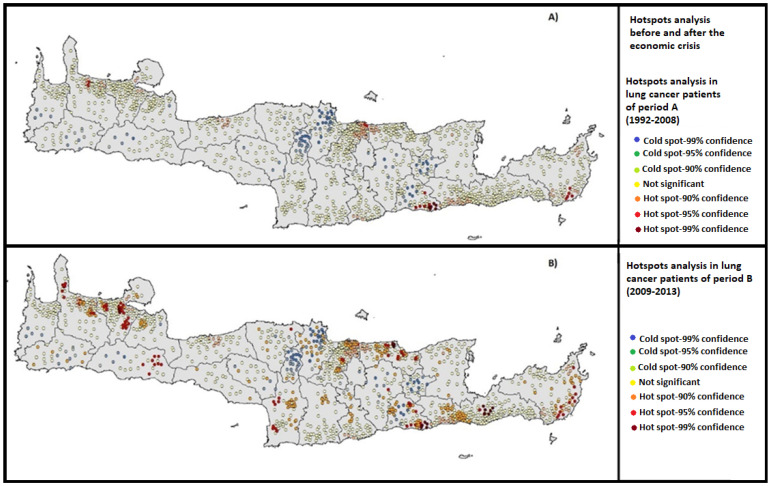
Hotspots analysis of LC cases in Crete: (**A**) before and (**B**) after the economic crisis, (*n* = 5057).

LC cases presented heterogeneous geographical distribution among and within the municipalities of Crete from 1992 to 2013. Detailed mapping of LC epidemiology has already been published by Sifaki-Pistolla et al. [21]. Figure 1 depicts the statistically significant LC hot spots (red and orange dots) according to the exact place of residence before (Figure 1A) and after (Figure 1B) the economic crisis. Several hot spots were observed before the economic crisis mainly in the capital city of Heraklion, in smaller regions of two other urban centers as well as in the south-east parts of the island. After the economic crisis (Period B), a significant increase in the observed hot spots was identified (*p* = 0.04). Statistically significant hot spots were visible in many sub-regions of the capital city and the other urban centers, as well as in several rural municipalities in south-east and north-east Crete. In addition, new hot spots were observed within municipalities that presented cold or no significant spots during period A (e.g., in south-west municipalities). 

### 3.3. Predictors and Overall Risk of LC Mortality

Multiple predictors of LC mortality were identified for the two under study periods and were presented in Table 2. The CCI%, the family medical history of LC or other lung disease, the personal history of LC or other lung disease, ever smokers and alcohol consumers, exposure to OAP, and the deprivation index were found to be significant predictors (*p* < 0.005) of increased LC mortality, when adjusting for age, gender, stage at diagnosis and place of residence. After 2009, the risk of LC mortality increased even more for smokers (RR = 5.7; 95%CI = 5.2–6.3) and those living in highly deprived geographical regions (RR = 5.4; 95%CI = 5.1–5.8). The CCI% and exposure to OAP were also much increased during period B (RR = 4.9; 95%CI = 4.4–5.5 and RR = 2.3; 95%CI = 1.9–2.8, respectively). 

**Figure 2:**
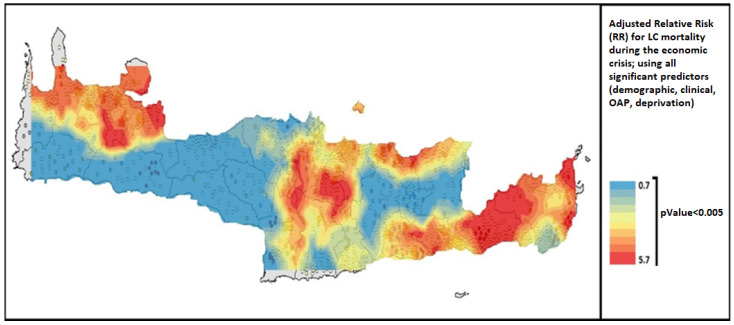
Risk for LC mortality during the economic crisis, testing for multiple significant predictors, (*n* = 3599).

Figure 2 presented the high-risk areas of increased LC mortality in Crete during the economic crisis, after testing for all significant predictors. The multiple effect of the eight predictors (Table 2) was higher (orange and red colors) in half of the east part of the island as well as in some northwest regions. The adjusted RR ranged from 0.7 to 5.7 within the island (*p* < 0.05).

## 4. Discussion

The current study managed to meet its main objectives and report some key findings on LC trends during the Greek economic crisis. The constantly changing LC trends in terms of incidence, mortality, and survival were among the major findings. The impact of the economic crisis was measured and found to be significant both for LC burden and the associated demographic, socio-economic, clinical, lifestyle, and environmental factors. In addition, various LC hot spots were identified in several geographical regions within the island after the onset of the economic crisis. Furthermore, several predictors of LC-increased mortality were found and contributed to the identification of the high-risk areas in Crete during the austerity period. These factors included multiple morbidities, smoking and alcohol history, exposure to OAP, and deprivation index. 

The global economic crisis has been associated with increased unemployment, reduced public-sector expenditure on healthcare, and an excessive increase in some non-communicable diseases, especially in mortality [31]. Lung cancer, as well as other types of cancer, is among the major causes of mortality in societies undergoing massive economic and structural changes [31,32]. The corresponding contribution of cancer to increased mortality during austerity or periods of instability were visible in many studies including a study in New Zealand [32]. Similarly, to the current study, significant disparities were found in terms of mortality and survival especially in females of low-income groups who tended to present rapidly increasing rates [32,33]. This observation might be attributed to several socioeconomic circumstances of disadvantage that women of low-income groups were more subjected to during the Greek economic crisis. Such a case is the use of biomass fuel for cooking and heating (with women spending many more hours inside the household compared to men) or the increase in smoking habits attributed to factors, such as an increased unemployment rates among women. Future research could explore the reasons behind this observation, as well as the rationale for the decrease in LC cases observed in men. Nevertheless, there are studies that haven’t found any profound increase in cancer mortality when exploring the short-term effects of economic declines [34]. This is most probable since they assess overall cancer mortality rather than cancer by type or site, while at the same time they focus on short-term exposure to economic instability (1–2 years) rather than longitudinal trends that could capture the dynamics of longer etiological periods of LC patients [10,34,35].

The present findings revealed that the age of patients at year of LC diagnosis increased from 68 years old prior to crisis to 77 years old during the financial crisis. In addition, an alarming increase was observed in regard to the LC stage at diagnosis. During the Greek economic crisis, more patients were diagnosed with LC at stage III or IV compared to prior to the economic crisis (44.8% and 17.2% vs. 39.7% and 13%, respectively). Similarly, a study in Romania (12) revealed a significantly higher percentage of patients diagnosed with late-stage breast cancer during the country’s economic crisis compared to the previous interval (56.3% vs. 46.9%). These findings are in accordance with the international literature, which supports that poverty is among the strongest predictors of LC since it significantly increases the risk of diagnosis at a higher age, or later stage, as well as the rapid increase in multiple morbidities [12,15,36]. Furthermore, economic deprivation may limit access to and utilization of continuous and high-quality medical care and, therefore, increase the risk of lower survival [37]. Multiple other factors seem to contribute to this excess risk especially in regions or population groups in which they have a joint effect [22]. Our findings are in accordance with previous reports, concerning certain risk factors of poor LC mortality, such as smoking [21,38], exposure to OAP [22,39], multiple morbidities [40], and economic deprivation [36,41,42].

It is evident that the recent reforms of the Greek National Health System and the measures imposed seem to have dubious long-term consequences for the Greek public health [5,43,44]. This is leading slowly to a health crisis even among chronic patients. LC patients are among the high-risk population groups since their incidence rated had started increasing even prior the fiscal crisis due to the constantly changing lifestyle habits [21,44]. After 2009, the trends seem to be more excessive even among women and rural inhabitants that due to high HPI and exposure to multiple risk factors present significantly lower survival. 

### Strengths and Limitations

This is the first study in Greece and Crete in particular that incorporates more than two decades of data on LC and the associated risk factors. Nevertheless, a number of limitations may exist. The fact that information on OAP exposure and the HPI index were expressed at a sub-regional level rather than at individual-level may have led to minor over-estimations of the overall risk of LC mortality. In addition, the role of tobacco smoking in LC incidence and mortality has already been acknowledge by the literature as of major importance and could insert several biases in LC epidemiological studies [35]. Therefore, studies should focus on adjusting or excluding the potential confounders including smoking and/or OAP or multiple morbidities. To this end, the current study attempted to manage these parameters and assess the overall risk of LC mortality through the Bayesian multivariate regression model.

Furthermore, it should be noted that misclassification, information, and systematic biases that usually exist in large cohorts or time-series were successfully minimized in the current study through the systematic and extensive quality controls of the CRC. As mentioned in the methods section, the high-quality rates of completeness = 98% and validity/accuracy = 97% could assure the overall value of the data. 

## 5. Conclusions

The increased LC burden after the onset of the economic crisis, along with a changing pattern of LC predictors and the excess risk in certain deprived geographical regions in Crete are among the main components of the added value of this study in the current literature. These reveal the urgent need of geographically oriented interventions and cancer control programs focusing on the most deprived or vulnerable population groups. The impact of the economic crisis on cancer patients and the effects to come during the austerity period stress the importance of expanding population-based, accessible, and comprehensive rehabilitation programs and bolstering of cancer prevention services. 

## Figures and Tables

**Table 1:** LC burden in Crete and the demographic, clinical, socioeconomic, and environmental profiles of the under-study population before and after the economic crisis, (*n* = 5057).

Characteristics	Lung Cancer Cases		Variation Rate from 1992 to 2013	*p* Value
	Period A (1992–2008)	Period B (2009–2013)		
**ASIR/100,000/year**	37.9	47.8	+9.8	0.01
**ASMR/100,000/year**	30.5	43.8	+13.3	<0.001
**5 year Net-Survival (%)**	14.6	12.1	−2.5	0.04
**Gender (%)**				0.01
*Males*	91.8	82.4	−9.3	
*Females*	8.2	17.6	+9.4	
**Age at year of diagnosis (Median)**	68	77	+9	<0.001
**Stage at year of diagnosis (%)**				<0.001
*I*	17.1	6.1	−11	
*II*	12.2	8.2	−7.9	
*III*	13	17.2	+8.2	
*IV*	39.7	44.8	+10.2	
*Not known*	18	18.1	+0.2	
**Adjusted Charlson Comorbidity Index-CCI% (Median)**	3	4	+1	0.04
**Family medical history of LC or other lung disease**				0.62
*No*	15.1	15.0	−0.1	
*Yes*	67.6	67.5	−0.1	
*Not known*	17.3	17.5	+0.2	
**Smoking status (%)**				0.02
*Never smoker*	19.9	12.8	−7.1	
*Ever smoker*	71.8	79.2	+7.4	
**Not known**	8.3	8	−0.3	
**Alcohol consumption (%)**				0.02
*No*	29.9	21.1	−8.8	
*Yes*	32	41.4	+9.4	
*Not known*	38.1	37.5	−0.6	
**Exposure to OAP (% of LC patients potentially exposed)**	25.1	34.9	+9.8	<0.001
**Deprivation Index (Median)**	17.5	29.2	+11.7	<0.001

**Table 2:** Multiple predictors of LC mortality before and after the economic crisis, (*n* = 3599).

Covariates	Period A (1992–2008)		Period B (2009–2013)	
	Relative Risk * of LC Death (95%CI)	*p* Value	Relative Risk * of LC Death (95%CI)	*p* Value
Adjusted Charlson’s Comorbidity Index-CCI%	4.1 (3.8–4.4)	0.02	4.9 (4.4–5.5)	0.04
Family medical history of LC or other lung disease	4.0 (3.5–4.6)	0.03	4.0 (3.3–4.8)	0.04
Ever smokers	4.8 (4.6–5.1)	0.01	5.7 (5.2–6.3)	0.03
Alcohol consumers	1.8 (1.2–2.4)	0.02	2.1 (1.3–3.0)	0.04
Exposure to OAP	1.9 (1.6–2.1)	0.02	2.3 (1.9–2.8)	0.03
Deprivation Index	1.5 (1.1–2.0)	0.03	5.4 (5.1–5.8)	0.03

* Model adjusted for age, gender, stage at diagnosis and place of residence.

## Data Availability

Available only for research purposes.
